# The Lipid Raft Proteome of African Trypanosomes Contains Many Flagellar Proteins

**DOI:** 10.3390/pathogens6030039

**Published:** 2017-08-24

**Authors:** Aabha I. Sharma, Cheryl L. Olson, David M. Engman

**Affiliations:** 1 Departments of Pathology and Microbiology-Immunology, Northwestern University, Chicago, IL 60611, USA; aabhasharma2012@u.northwestern.edu (A.I.S.); c-olson@northwestern.edu (C.L.O.); 2 Department of Pathology and Laboratory Medicine, Cedars-Sinai Medical Center, Los Angeles, CA 90048, USA

**Keywords:** trypanosomes, lipid rafts, flagellum, intraflagellar transport

## Abstract

Lipid rafts are liquid-ordered membrane microdomains that form by preferential association of 3-β-hydroxysterols, sphingolipids and raft-associated proteins often having acyl modifications. We isolated lipid rafts of the protozoan parasite *Trypanosoma brucei* and determined the protein composition of lipid rafts in the cell. This analysis revealed a striking enrichment of flagellar proteins and several putative signaling proteins in the lipid raft proteome. Calpains and intraflagellar transport proteins, in particular, were found to be abundant in the lipid raft proteome. These findings provide additional evidence supporting the notion that the eukaryotic cilium/flagellum is a lipid raft-enriched specialized structure with high concentrations of sterols, sphingolipids and palmitoylated proteins involved in environmental sensing and cell signaling.

## 1. Introduction

Cell membranes constitute a large fraction of the biomass of a typical cell and contain 25–40% of all cellular proteins [1]. Because of the hydrophobic environment of the lipid bilayer, membrane proteins require membrane spanning α-helices or β-sheets or hydrophobic lipid modifications to reside in this environment. The lipid modifications include glycosylphosphatidylinositol anchors, N-terminal myristic acid tails, palmitic acid tails acquired through cysteine acylation, and C-terminal prenyl groups. Within cell membranes are microdomains called lipid rafts, which are liquid-ordered (gel-like) regions containing high concentrations of 3-β-hydroxysterols, sphingolipids and proteins having acyl modifications such as myristoylation and palmitoylation [2,3,4,5]. Lipid rafts are present in all eukaryotic cells and provide platforms for the assembly of cell-signaling complexes [2,6,7]. Lipid rafts have the special property of detergent resistance and can be extracted from the membrane using cold non-ionic detergents [6]. Lipid rafts or raft-associated proteins are involved in the pathogenesis of a number of human diseases, including infectious diseases [8]. 

In kinetoplastids, single-celled organisms containing a unique mitochondrial DNA called kinetoplast DNA, lipid rafts and raft-associated proteins have been characterized in *Leishmania* spp. [9], *Trypanosoma cruzi* [10,11] and *Trypanosoma brucei* [4,12]. The intracellular trypanosomatids *T. cruzi* and *Leishmania* exploit lipid rafts for immune evasion and host cell infection [7]. Cholesterol is essential for the pathogenesis of malaria and leishmaniasis both by promoting the attachment of the parasite to the host cell and by catalyzing the internalization of the parasite into the host cell [13]. *Leishmania* can survive and proliferate inside macrophages by disrupting lipid rafts, causing an alteration in CD40 signaling leading to defective antigen presentation resulting from increased membrane fluidity [14]. Similarly, *T. cruzi* recruits lysosomes and forms ceramide-enriched endocytic vesicles in a calcium-dependent manner to facilitate its entry into the host cell [13]. Thus, the dynamics of host lipid rafts and parasite lipid rafts has important implications in signaling events leading to parasite infection. 

*T. brucei*, the causative agent of human African trypanosomiasis (sleeping sickness), is an elegant model organism for studies of basic eukaryotic cell biology and biochemistry. Both BSF and PF stages are genetically manipulable by both RNA interference [15] and CRISPR-Cas9 genome editing [16]. The life cycle of this parasite involves several stages in the tsetse fly insect vector and the bloodstream of the mammalian host. Two GPI-anchored proteins, variant surface glycoprotein in the bloodstream form (BSF) and procyclin in the insect mid-gut form, the procyclic (PF), dominate the surface of *T. brucei*, but are not lipid raft associated due to unique properties of the GPI anchor structure in trypanosomes [17]. 

Work done by our laboratory and others has revealed the flagellar membrane of *T. brucei* to be highly enriched in sterols, sphingolipids and dually-acylated proteins [4,11,18]. Phospholipids are also enriched in the flagellar membrane [11,19]. Although there are clear differences in the sterol, sphingolipid and dually acylated protein compositions of *T. brucei* flagellar and cell body membranes, the set of proteins that are associated with lipid rafts in trypanosomes and other protozoans is not known. In the current study, we determined the lipid raft proteome of *T. brucei* PF cells and discuss the findings in the context of flagellar proteins.

## 2. Results

**Figure 1:**
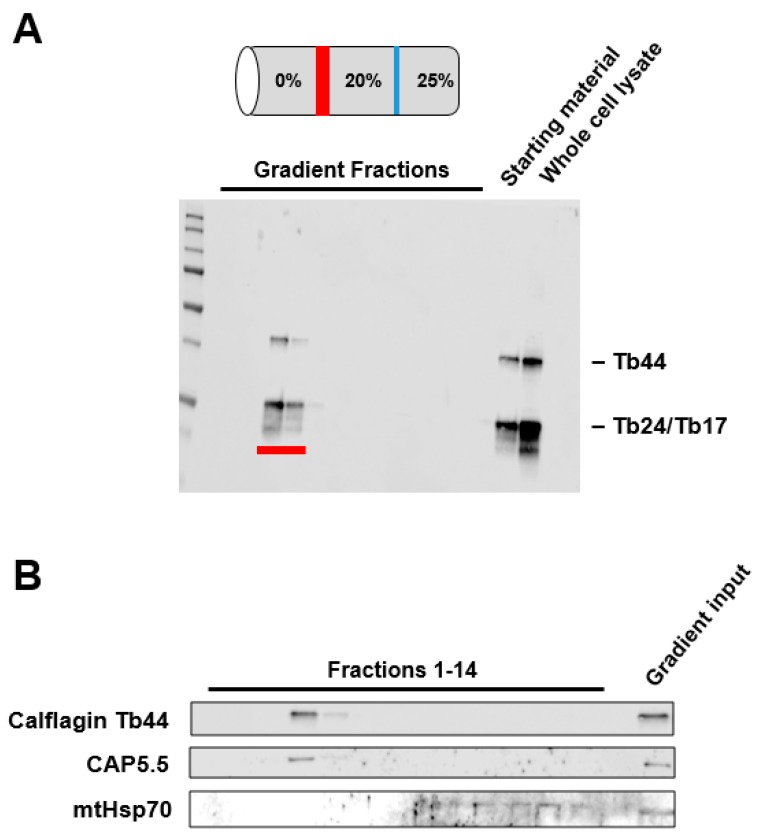
Purification and characterization of *Trypanosoma brucei* lipid rafts. (**A**) *T. brucei* PF lipid rafts were prepared as described in Methods and subjected to Optiprep step gradient ultracentrifugation (diagrammed at top). Buoyant fractions containing lipid rafts floated to the top interface (red). Western blot analysis of fractions was performed using antibodies specific for the raft protein Calflagin (Tb44, Tb24 and Tb17) and the two fractions containing Calflagin (red) were used for proteomics; (**B**) Optiprep gradient fractions were analyzed by western blotting with positive control (Calflagin Tb44, CAP5.5) and negative control (mtHsp70) antibodies.

We isolated lipid rafts from PF *T. brucei* using a detergent-free method [20] that has been employed previously for similar studies in other organisms [21,22,23]. The lipid raft preparation was applied to a discontinuous Optiprep gradient and gradient fractions were analyzed by SDS-PAGE and western blotting for Calflagin, a well-characterized family of three flagellar calcium binding proteins (Tb44, Tb24 and Tb17) that associate with lipid rafts [4] via dual acylation with myristate and palmitate [12] (Figure 1A). The lipid raft fractions also contain the raft-associated protein CAP5.5 [24] but do not contain mitochondrial Hsp70 (mtHsp70), which is found in the mitochondrial matrix (Figure 1B).

### 2.1. Trypanosome Lipid Raft Proteome

The lipid raft proteome was determined as described in Methods. While known lipid raft-associated proteins such as Calflagin and CAP5.5 were present in this proteome, the GPI-anchored major surface protein procyclin was absent, as expected, since it does not associate with lipid rafts [4]. After filtering out contaminants, 351 proteins remained in the lipid raft protome (Supplementary Table S1). Proteins that are indicated in **bold text** are of special interest and include positive controls Calflagin and CAP5.5. Eighteen percent of the 351 proteins are of unknown function. Proteins having known or likely functions include several putative flagellar-signaling molecules, including Rab-like GTPases that are part of the intraflagellar transport protein (IFT) complex-B. These include IFT 22 (RabL5) and IFT27 (RabL4), Rab-like small G proteins with possible roles in IFT regulation [25,26]. Arginine kinase, a flagellar protein important for infection in tsetse flies [27] is also present, as is Aquaporin, a plasma membrane-specific surface drug transport protein [28] involved in pentamidine resistance in BSF parasites [29]. 

### 2.2. Comparison of the Lipid Raft Proteome with the Flagellar Proteome, GO Terms and Protein Localization

**Figure 2:**
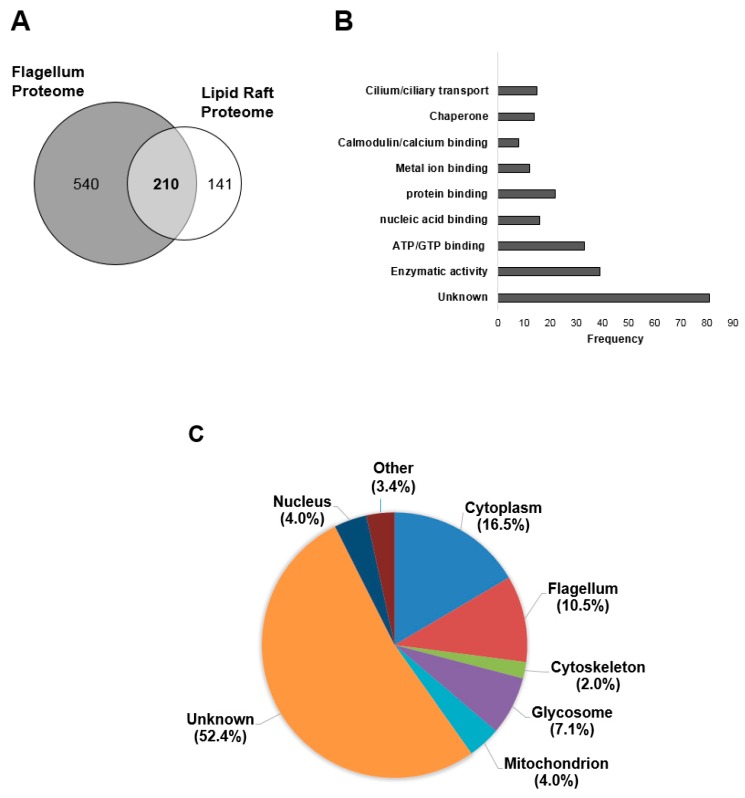
The lipid raft proteome of *Trypanosoma brucei* is highly enriched in flagellar proteins. (**A**) Intersection the PF lipid raft proteome with the PF flagellar proteome [30]. 60% (210/351) of the raft proteins are flagellar; (**B**) Bar graph representing the most common GO terms among PF lipid raft proteins; (**C**) Pie chart representing the most common localizations of the proteins found in the PF lipid raft proteome.

Lipid rafts are known to contain proteins having acyl modifications such as myristoylation or palmitoylation [2,3,4,5]. Additionally, recent work from our group [4,18] and others [19] on lipid rafts of both the PF and BSF cells have highlighted components of lipid rafts such as sterols and sphingolipids to be enriched in the flagellar membrane of *T. brucei*. Thus, we further analyzed the PF lipid raft proteome by determining its intersection with the membrane intact total flagellar proteome published by Subota et al. [30] (Figure 2A).

Lipid rafts are known to contain proteins having acyl modifications such as myristoylation or palmitoylation [2,3,4,5]. Additionally, recent work from our group [4,18] and others [19] on lipid rafts of both the PF and BSF cells have highlighted components of lipid rafts such as sterols and sphingolipids to be enriched in the flagellar membrane of *T. brucei*. Thus, we further analyzed the PF lipid raft proteome by determining its intersection with the membrane intact total flagellar proteome published by Subota et al. [30] (Figure 2A). 210 (~60%) of the proteins in the PF lipid raft proteome are also present in the in PF flagellar proteome and mostly include non-contaminating flagellar proteins. This is an astonishing enrichment of flagellar proteins given that the cilium/flagellum contains only a small fraction (~10%) of total cellular proteins [31]. Several of these proteins included putative and confirmed palmitoylated proteins (Supplementary Table S1), suggesting an enrichment of flagellar palmitoylated proteins in the PF lipid raft proteome. When GO terms of the proteins found in the PF lipid raft proteomes were analyzed for enrichment, enzymatic activities, ATP/GTP binding activities, protein binding and cilium related functions displayed particular enrichment (Figure 2B, Supplementary Table S1). 23% of the proteins are of unknown function and thus did not have associated GO terms.

When we compiled the published localizations of the proteins found in the lipid raft proteome, most had not been localized (Supplementary Table S1). Among the proteins that had been localized, the majority are cytoplasmic. Consistent with the lipid raft-flagellum intersection analysis, flagellar proteins are particularly abundant (Figure 2C). The majority of the proteins that have cytoplasmic locations also have flagellar annotations based on the literature (Supplementary Table S1). Several mitochondrial and glycosomal proteins are in the lipid raft proteome as well (Figure 2C).

### 2.3. Testing Detergent Resistant Membrane Association of Selected Lipid Raft Proteins

**Figure 3:**
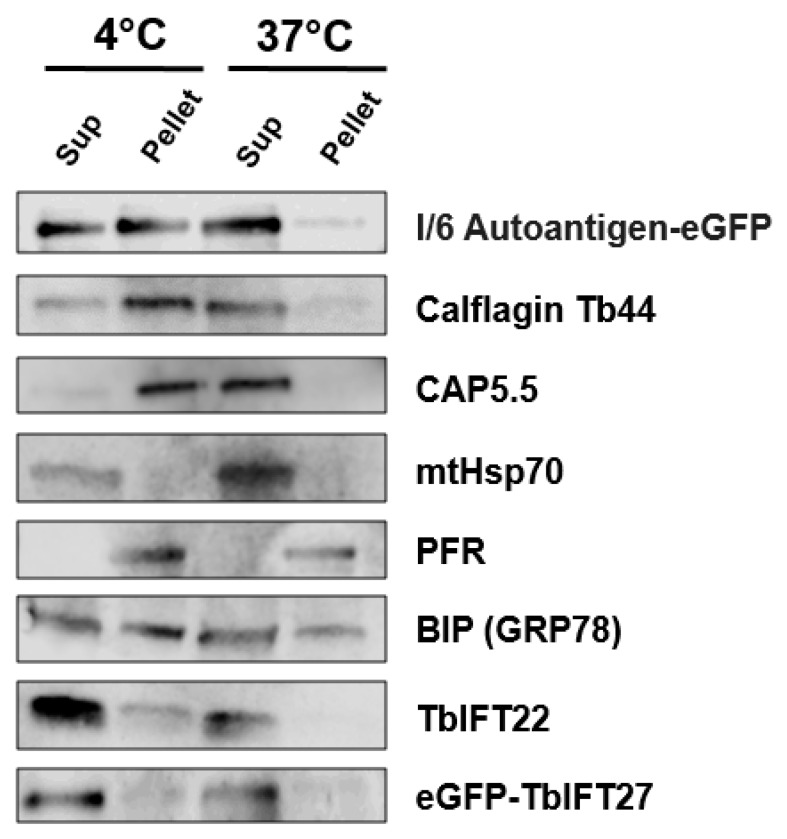
Association of lipid raft proteins with detergent resistant membranes. Normal PF cells or those expressing autoantigen I/6-eGFP or eGFP-IFT27 were treated with 1% Triton-X at either 4 °C or 37 °C and separated into either soluble supernatant (Sup) or insoluble (Pellet) fractions via centrifugation. Fractions were analyzed by western blotting to determine DRM association, with proteins found in the pellet at 4 °C but the supernatant at 37 °C being in DRMs. Most proteins were detected with specific antisera, except for autoantigen I/6-eGFP and eGFP-IFT27, which were detected using anti-GFP. The following controls were included: Calflagin Tb44 and CAP5.5 are known DRM-associated proteins, mtHsp70 is a detergent soluble mitochondrial matrix protein, paraflagellar rod 2 protein (PFR) is a detergent insoluble cytoskeletal protein, and BIP (GRP78) can be considered a “loading control” since it is found in all fractions.

One protein that is particularly abundant in the lipid raft proteome is autoantigen I/6 (Tb927.7.3440), a cytoskeletal protein that is highly immunogenic and possesses a non-functional EF-hand [32,33]. Not much more is known about this protein, but it is one of the high confidence palmitoylated proteins we determined in a previous study [34], and thus an excellent candidate for lipid raft association. We found that autoantigen I/6 is associated with DRMs using a standard assay in which DRM proteins are found in the pellet at 4 °C but become solubilized upon warming to 37 °C (see Methods). Since we did not have an I/6-specific antibody, we employed a cell line expressing autoantigen I/6 fused to e-GFP. A large fraction of autoantigen I/6-eGFP was detected in the pellet fraction at 4 °C and in the soluble fraction upon warming to 37 °C (Figure 3). Positive controls Calflagin and CAP5.5 behaved similarly in this assay. mtHsp70 and paraflagellar rod 2 protein (PFR) were included as the detergent soluble and insoluble controls, respectively, and BIP (GRP78) was included because it is found in all fractions. 

We were initially surprised to find so many IFT proteins in the lipid raft proteome and therefore decided to test the DRM association of two Rab-like IFT proteins—TbIFT22 and TbIFT27. TbIFT22 was abundant in an initial BSF lipid raft proteome (data not shown), and also had several peptides each in two biological replicates in the PF lipid raft proteome (Supplementary Table S1). We were able to test the DRM association of TbIFT22 using a specific antibody kindly provided by the Bastin lab. We did not have an TbIFT27-specific serum, so we generated a cell line expressing eGFP-TbIFT27 for this analysis. Both IFT proteins displayed modest DRM association, being found in both the 4 °C pellet and supernatant, but solely in the supernatant upon warming to 37 °C (Figure 3). 

## 3. Discussion

Lipid rafts are liquid-ordered membrane microdomains that contain high levels of 3-β-hydroxysterols, sphingolipids and proteins frequently having acyl modifications such as myristoylation or palmitoylation [2,3,4,5]. They are present in all eukaryotic cells and provide platforms for the assembly of cell-signaling complexes in eukaryotes [2,6,7]. Here, we describe the procyclic form (PF) lipid raft proteome of *T. brucei*, which is highly enriched in proteins found in the flagellum. There were several putative signaling proteins and proteins implicated in the virulence of BSF parasites. Surprising revelations included the presence of several IFT proteins, which have not been studied in the context of lipid rafts.

We were able to test the DRM association of a few proteins found in our procyclic lipid raft proteome. *T. brucei* autoantigen I/6 protein, which is recognized by self-reactive antibodies from uninfected mice, is a putative palmitoylated protein [34] detected in our lipid raft proteome. Using the 4/37 DRM assay, we were able to confirm its association with DRMs (Figure 3). This protein is also known to be associated with the cytoskeleton [32], as is CAP5.5 [24]. Further work is necessary to confirm if it uses the palmitoylation modification to associate with the lipid rafts and the role played by this protein in the biology of *T. brucei*. 

One of the more surprising revelations from the lipid raft proteome is the presence of several IFT proteins. The PF raft proteome contained TbIFT88, TbIFT27, TbIFT22 and other IFT components. TbIFT22 and TbIFT27, both of which are Rab-like proteins, are of particular interest. TbIFT22 was present in two out of three PF raft proteome replicates but was eliminated from the final list due to too few peptide hits. This could be due to its relatively lower abundance. TbIFT22 is essential for flagellum assembly in *T. brucei* [35]. TbIFT27 is a small GTPase essential for cargo loading for IFT [26]. While IFT27 is partly associated with the DRM fraction, a notable proportion of IFT22 associated to the DRM (Figure 3). These Rab-like proteins could either have been detected while interacting with a raft associated protein during their transport process or could require lipid rafts to carry out their function. Several small GTPases such as Rac and Rho studied in the context of mammalian cells are known to associate with lipid rafts [36,37,38]. Therefore, further exploration of IFT27 and IFT22 in the context of lipid rafts could reveal more about the connection relationship between IFT and ciliary lipid rafts. Given that the ciliary membrane is enriched in components of lipid rafts [4], it would not be surprising to find a role for lipid rafts in IFT. 

The lipid raft proteome of the PF *T. brucei* also contained several calcium-signaling and transport proteins besides Calflagin. TbCALP1.1 and TbCAP5.5 (TbCALP4.1), the calpains that are part of the family of calcium dependent cysteine proteases expanded in kinetoplastids [39], were detected in the PF lipid raft proteome. CAP5.5 has the dual acylation motif needed for membrane association [40]. TbCALP1.1 is a soluble flagellar protein with an enrichment at the flagellar tip [41]. Surprisingly, CALP1.6 and 7.2, which are both present at higher transcript levels in PF [40] were not detected in the PF raft proteome. Since the exact functions of different calpains and the roles played by lipid rafts in their functions have not yet been characterized, functional characterizations of calpains could unravel not just the evolutionary reason for their expansion in kinetoplastids but also the significance of their association with lipid rafts.

Another protein of interest includes iron superoxide dismutase (Tb927.11.15910, previously Tb11.01.7550), a glycosomal protein commonly referred to as SODB1 [42]. Depletion of SODB causes a growth defect [42]. Overexpression of its homolog in *T. cruzi*, Fe-SOD, results in higher susceptibility to trypanocidal drugs benznidazole and gentian violet [43]. Although the role of lipid rafts in SOD function has not been investigated in trypanosomes, a Cu/Zn SOD in *Cryptococcus neoformans*, which is an antioxidant virulence factor, is concentrated in lipid raft membranes [44]. Lipid rafts are hypothesized to serve as hubs that cluster virulence factors [44]. Thus, further work investigating proteins such as SODs in the lipid raft proteome could reveal several other virulence factors that could be potential drug targets. 

We previously discovered that electron-dense DRM particles are specifically associated with the flagellar axoneme [4] and some s have hypothesized that these particles may be platforms for the assembly of cell sensing and signaling complexes [45,46]. The collective results presented here strongly support this hypothesis. The PF lipid raft protein contains many flagellar proteins and, specifically, many IFT proteins and virulence factors. Further characterization of individual proteins is necessary to confirm their associations with the lipid rafts and the importance of lipid raft association in their functions.

Limited study of lipid raft proteomes in most eukaryotic pathogens challenges our ability to compare the *T. brucei* lipid raft proteomes with other pathogens or with ciliary lipid raft proteomes. The only known lipid raft proteome for a eukaryotic pathogen has been published for the apicomplexan parasite *Plasmodium falciparum* [47]. This proteome has similarities in the types of proteins found in the *T. brucei* lipid raft proteome. Both of the proteomes have long lists of proteins of unknown functions and proteins involved in protein binding and signaling. Our lipid raft proteome contains GO term profiles similar to those of raft proteomes of other eukaryotic cell types, including *Caenorhabditis elegans* and the unicellular phytoplankton *Emiliania huxleyi* [48,49,50,51]. Future determination of the lipid raft proteomes of cells containing primary cilia or flagella will allow us to further elucidate the important roles played by cilia in sensing and signaling.

Further study of the lipid rafts in the flagellum of a kinetoplastid such as *T. brucei* not only should elucidate the specific raft components required for pathogenesis but also should reveal conserved signaling pathways initiated through the cilia of higher eukaryotes. Previous lipidomic analyses of *T. brucei* have shown this organism to be rich in major classes of glycerophospholipids including phosphatidylcholine, phosphatidylethanolamine, phosphatidylserine, and phosphatidylinositol, and sphingolipids, which also play vital roles in the biology of higher eukaryotes [52]. Such similarities in lipid usage between trypanosomatids and higher eukaryotes including mammalian cells can be exploited through the study of lipid synthetic pathways in simpler trypanosome model system. Moreover, the high abundance of lipid rafts in the trypanosome ciliary membrane allows for the study of ciliary-signaling pathways conserved in higher eukaryotes in a much simpler model system. Precise differences in the identities and/or concentrations of membrane lipids and lipid raft proteins of trypanosomatids are yet to be unveiled. Future studies focusing on ciliary lipid rafts, the raft associated putative proteins identified in our work and their roles in environmental and life cycle stage specific functions in signaling and virulence would provide major contributions to our understanding of trypanosomatid pathogenesis and establishment of the cilium as a general signaling organelle in eukaryotes. 

## 4. Materials and Methods

### 4.1. Parasites

*Trypanosoma brucei* PF 29-13 cells originally derived from Lister strain 427 [53] were cultured at 27 °C in SDM-79 medium [54] supplemented with 10% dialyzed FBS (Sigma-Aldrich, Saint Louis, MO, USA), 7.5 μg/mL hemin, 100 U/mL penicillin/streptomycin, 50 μg/mL hygromycin, and 15 μg/mL G418. 

### 4.2. Lipid Raft Isolation for Raft Proteome

Lipid rafts were isolated using a detergent-free method of MacDonald and Pike [20]. 8 × 10^8^ cells of 29-13 PF *T. brucei* were pelleted by centrifugation at 1000× *g*. Cells were washed twice with PBS supplemented with 13 mM glucose (PBSG) and suspended at 8 × 10^8^/mL in base buffer (20 mM Tris-HCl, pH 7.8, 250 mM sucrose) supplemented with 1 mM CaCl_2_, 1 mM MgCl_2_, and protease inhibitors. All of the following procedures were carried out in a cold room. Cells were lysed by ten passages through 1.5 inch long 22 ga needle and centrifuged at 1000× *g*. The supernatant was collected and the pellet was lysed again and centrifuged and the second supernatant was collected and combined with the first. The combined supernatant fractions were subjected to Optiprep gradient (0–25%) ultracentrifugation at 52,000× *g* using an SW41Ti rotor in a Beckman ultracentrifuge for 90 min as described previously [20]. 800 μL fractions, including the buoyant proteins at the upper gradient interface, were run on an SDS-PAGE gel and transferred to nitrocellulose membranes for western blot analysis. Calflagin [55] and CAP5.5 (generously provided by the Gull lab) were used as positive controls and mtHsp70 [56] and PFR2 (generous gift of the Hill lab) as negative controls for the blot. Fractions corresponding to buoyant lipid rafts were frozen at −80 °C until ready for proteomic analysis. 

### 4.3. Proteomics and Data Analyses on Lipid Raft Associated Proteins

A BCA assay (Thermo Fisher Scientific, Waltham, MA, USA) was used to determine the protein concentration of the raft fractions. Lipid raft fractions equivalent to 30 μg of protein from four different biological replicates were run on 10% SDS-PAGE gel to get rid of contaminants and Optiprep before submitting the full gel lanes for proteomic analysis. The steps that follow were performed at the Northwestern University Proteomics Core. Each gel lane was cut into three parts based on molecular weight before performing bottom-up proteomic using a protocol described previously [57]. Gel slices were washed in 100 mM ammonium bicarbonate/acetonitrile (AmBic/ACN) and reduced with 10 mM dithiothreitol at 50 °C for 30 min. Cysteine was alkylated with 100 mM iodoacetamide in the dark for 30 min, room temperature. The slices were then washed in 100 mM AmBic/ACN prior to digestion with 500 ng trypsin overnight at 37 °C. Supernatants containing peptides were saved into a new tube. Gel slices were washed at room temperature for ten minutes with gentle shaking in 50% ACN/5% formic acid (FA), and supernatant was saved to peptide solution. The washes were repeated each with by 80% ACN/5% FA, and 100% ACN, and all supernatants were saved into peptide solution then dried by speedvac. After lyophilization, peptides were reconstituted with 0.1% FA in water and injected onto a trap column (150 µm ID × 3 cm) coupled with a nanobore analytical column (75 µm ID × 10 cm, both ReproSil C18aq, 1.9 µm). Samples were separated using a linear gradient of solvent A (95% water, 5% acetonitrile, 0.1% formic acid) and solvent B (5% water, 95% acetonitrile, 0.1% formic acid) over 200 min. MS data were obtained on a Velos Orbitrap (Thermo Fisher Scientific) mass spectrometer. Data were searched using Mascot (Matrix Science, Boston, MA, USA) 2.5 against the UniProt database, *T. brucei*, and results were reported at 1% FDR (false discovery rate) using Proteome Discoverer software 1.4 (Thermo Fisher Scientific). Raw data obtained from the core were refined by removing non-specific hits that did not correspond to the protein size range for the gel piece in question. 

### 4.4. Intersection of Different Proteomes with Lipid Raft Proteome

The PF flagellar proteome published by Bastin [30] and the procylic palimtoylome published by our group [34] were downloaded from the Tritryp database [58]. This allowed consistency of accession numbers for intersection analysis. Microsoft Access 2013 was used to perform intersection queries. When conducting intersection analysis, only proteins with at least 2 peptide hits in a given proteome were included to reduce background. 

### 4.5. Detergent Resistant Membrane Preparation

1 × 10^7^ PF *T. brucei* cells expressing eGFP-TbIFT27 or autoantigen-eGFP were treated with 1% Triton-X at either 4 °C or 37 °C and separated into either soluble supernatant or insoluble pellet fractions via centrifugation at 1000× *g* to separate the DRM fractions. GFP specific-chicken serum was used to detect eGFP-IFT27 and autoantigen I/6-eGFP (1:10,000) (Aves Labs, Tigard, OR, USA). Other proteins were detected using specific antisera (dilution): TbIFT22 (1:500) (Bastin Lab, Paris, France), Calflagin (1:1000), PFR2 (1:1500) (Hill lab, Los Angeles, CA, USA), BIP (1:1000) (Bangs lab, Buffalo, NY, USA).

## Supplementary Materials

The following are available online at www.mdpi.com/2076-0817/6/3/39/s1, Table S1: The *Trypanosoma brucei* Lipid Raft Proteome.
